# S1P Generation by Sphingosine Kinase-2 in Recruited Macrophages Resolves Lung Inflammation by Blocking STING Signaling in Alveolar Macrophages

**Published:** 2021

**Authors:** Jagdish C Joshi, Bhagwati Joshi, Ian Rochford, Dolly Mehta

**Affiliations:** Department of Pharmacology and Center for Lung and Vascular Biology, the University of Illinois at Chicago, Chicago, IL 60612, USA

**Keywords:** SPHK2, Lung injury, Macrophages, STING, ARDS

## Abstract

Acute respiratory distress syndrome (ARDS) is the major cause of mortality among hospitalized acute lung injury (ALI) patients. Lung macrophages play an important role in maintaining the tissue-fluid homeostasis following injury. We recently showed that circulating monocytes recruited into the alveolar space suppressed the stimulator of type 1 interferon genes (STING) signaling in alveolar macrophages through sphingosine-1-phosphate (S1P). We used CD11b-DTR mice to deplete CD11b^+^ monocytes following LPS or *Pseudomonas aeruginosa* infection. Depletion of CD11b^+^ monocytes leads to the persistent inflammatory injury, infiltration of neutrophils, activation of STING signaling and mortality following lung infection. We demonstrated that adoptively transferred SPHK2-CD11b^+^ monocytes into CD11b-DTR mice after pathogenic infection rescue lung inflammatory injury.

## Author Commentary

Acute lung injury (ALI) is associated with bacterial and viral infection such as *Pseudomonas aeruginosa*, SARSCOV2 virus, sepsis or drug toxicity which leads to acute respiratory distress syndrome (ARDS), predominantly in elderly patients. ARDS compromises survival of 40% of the patients but the cellular and molecular basis leading to ARDS remains unclear. Macrophages are crucial for a coordinated response to pathogenic insult and to the restoration of tissue homeostasis after triggering inflammatory signaling [1,2]. During any pathogenic infection, macrophages initiate host-defence by generation of pro-inflammatory cytokine and recruitment of neutrophil by activating transcription factor NF-κB via cell-surface toll-like receptor 4 [3,4]. Macrophages also suppress the inflammatory signaling pathway in a time dependent manner which is essential to restore tissue homeostasis. Thus, macrophages play a dual role by both initiating and suppressing inflammatory signaling and reinstating tissue-homeostasis [5, 6].

In the lung, two major subsets of macrophages are known to exist; the alveolar macrophages (AMφ), identified by expression of surface markers CD11c^+^/SiglecF^+^, while the other population, known as recruited macrophages originated from monocytes during injury and are identified as being CD11b^+^/SiglecF^−^ [7–9].

AMφ trigger inflammatory signaling by releasing inflammatory cytokines and recruitment of neutrophils [10–13]. However, protracted inflammatory signaling from AMφ can itself damage vascular barrier function [13,14].

We showed that depletion of the recruited macrophage population, using diphtheria toxin (DT) in DTR-mice, during pathogenic insults induced proliferation and expansion of the inflammatory AMφ in the alveolar space [7]. We have shown that in CD11b^+^ monocytes depleted lungs, AMφ generated markedly higher IL6, IL-1β as well as IFN-β leading to the irreversible loss of lung vascular barrier function and rapid lethality of DTR mice. We demonstrated that, during lung injury, CD11b^+^Mφ alter the alveolar niche to cause AMφ to become anti-inflammatory, thereby preventing sustained inflammatory lung injury [7]. Our study demonstrated that adoptive transfer of CD11b^+^ monocytes from WT murine bone marrow into injured Mφ^dep^ mice rescued the anti-inflammatory function of AMφ and reversed lung vascular injury. Studies showed that monocytes can differentiate into AMφ [12] and CD11b^+^ CD11c^+^ macrophages in the lung represent a transitional pre-AMφ lineage [15,16]. Thus, an unanswered question is whether recruited CD11b^+^Mφ take on the AMφ signature and function after recruitment into the air space.

**Figure 1:**
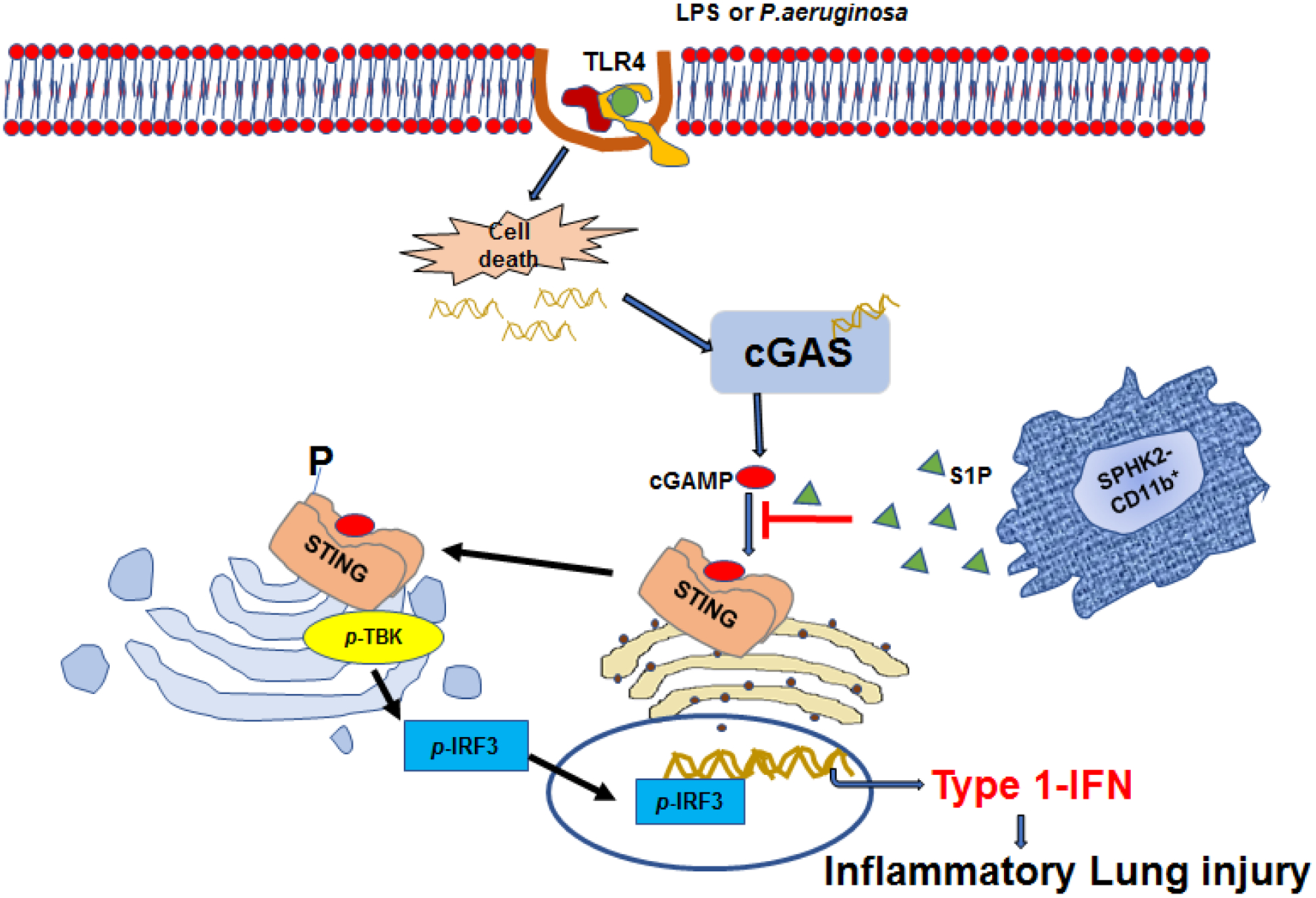
Upon recognition of LPS or Gram -ve bacteria like *Pseudomonas aeruginosa* by TLR4, there is cell death, which leads to the generation of dsDNA. This ds DNA is sensed by the DNA-sensing receptor cyclic GMP-AMP synthase (cGAS) which acts as substrate to catalyze the formation of cGAMP, which is a second messenger to activate STING. Activated STING dimerizes and translocate from the ER to the perinuclear region via Golgi to recruit and promote phosphorylation of TBK1-IRF3, which subsequently drives translocation of *p*-IRF3 to the nucleus to produce type 1-IFN. The SPHK2-CD11b+ recruited macrophages release SIP which compete with cGAMP and inhibit binding of cGAMP to STING, thus blocks the translocation of STING and phosphorylation of TBK1 and IRF3 and generation of type 1-IFN.

Upon sensing pathogens, cytokines released from AMφ can modulate the death of lung parenchymal cells depending on insult and its timing [17,18]. STING is a transmembrane homodimer located in the ER (endoplasmic reticulum) membrane and activated upon binding of the second messenger, cyclic GMP-AMP (cGAMP) [19–21]. DNA liberated upon cell death is sensed by cGAS [22,23], which thereafter converts GMP and AMP into the second messenger cGAMP to activate STING [20]. Upon activation STING translocate to the perinuclear region, Golgi body and binds and phosphorylate to activates TANK-binding kinase 1 (TBK1) and IFN regulatory factor 3 (IRF3). The phosphorylated IRF3 translocate to the nucleus and induces the generation of Type 1-IFN (Figure 1) [21,24]. We showed that depletion of CD11b^+^Mφ transiently increased cGAMP generation in control lungs while causing persistent increase in cGAMP levels in Mφ^dep^ lungs, resulting in enhanced STING signaling in these mice [7]. These findings indicate that recruited CD11b^+^Mφ in the airspace tightly control cGAMP-STING signaling in AMφ [7]. Macrophages are plastic in nature and can acquire either an inflammatory or anti-inflammatory phenotype depending on the inflammatory conditions [1]. The adoptive transfer of CD11b monocytes from WT or STING^−/−^ mice into Mφ^dep^ mice reversed the AMφ inflammatory signaling. Also, STING null mice failed to exhibit the lung edema and characteristic inflammatory signaling as observed in control mice [7]. However, a caveat of this study was that it did not show the extent to which STING null CD11b^+^ macrophages facilitated anti-inflammatory function of existing AMφ or induce differentiation of Cd11b^+^Mφ into AMφ [7].

In searching of the mechanism by which CD11b^+^Mφ suppressed STING signaling in AMφ, we focused on their paracrine role. Sphingosine-1-phosphate (S1P) generated by sphingosine kinases (SPHK1 and SPHK2) is a well-known bioactive lipid that protects vascular injury in various murine models of lung injury [25–27]. We showed that SPHK2 expression was higher in CD11b^+^Mφ than AMφ. Further, we showed that SPHK2 was required for maintaining SPHK activity and S1P levels in macrophages following LPS injury [7]. Adoptive transfer of WT-CD11b^+^ bone marrow monocytes blocked AMφ STING activity in Mφ depleted mice while transfer of SPHK2-deficient CD11b^+^ bone marrow monocytes failed to do so. In this case, AMφ remained inflammatory and led to non-resolvable lung injury. We also showed that SPHK2, but not SPHK1, was required for LPS stimulated S1P generation and that inhibition of SPHK2 kinase activity enhanced STING signaling in WT-BMDM as well as human macrophages. AMφ and CD11b^+^Mφ sorted from naïve WT and SPHK2-null lungs by flow cytometry were also assessed for SPHK1 and SPHK2 protein expression and differences in SPHK2 activity in CD11b^+^Mφ versus AMφ. Interestingly, we found that SPHK2 activity was 2-fold higher in CD11b^+^Mφ compared to AMφ whereas SPHK1 was expressed to the same extent in both macrophage subsets. Whereas the SPHK activity and S1P generation in macrophages isolated from bronchoalveolar lavage (BAL) of WT or *Sphk2−/−*mice were significantly reduced after LPS challenge in macrophages during peak of injury [7]. In human macrophages (U937 cell line), inhibition of SPHK2 activity using specific SPHK2 inhibitor, ABC294640 [28,29] increased TBK1, IRF3 and NF-κB-p65 phosphorylation. These findings suggested that recruited CD11b^+^Mφ functioned in a SPHK2 dependent manner.

Evidence indicates that activation of plasmalemmal S1P receptors respond to S1P generated by SPHK1 [25,26,30], whereas SPHK2-mediated S1P generation regulates cellular functions in a localized manner [31,32]. We showed that SPHK2 suppressed STING redistribution, indicating compartmentalized action of SPHK2 in targeting ER-STING signaling [7]. Consistent with this C-terminus of STING to induce STING signaling [33]. To assess S1P interaction with C-terminus of STING we used lysates from HEK cells transducing HA-full-length STING (1–379 aa) or the C-terminus of STING (HA-STING-CTD: aa149–379-STING). While CTD-STING was expressed to a lower extent than FL-STING in HEK, it did interact with S1P. Molecular docking predicted that S1P can competitively inhibit binding of cGAMP to STING C-terminal domain and thereby blocks down stream signaling [7]. However, several questions remain unanswered. First, how SPHK2 blocked STING translocation to Golgi? We expect that SPHK2 generated S1P might modulate the STING dimerization and making it unavailable for translocation. Second, what is the affinity of S1P towards STING in absence or presence of cGAMP and what are these binding sites? In order to check the binding affinity of S1P to STING, cGAMP binding and other possible sites in STING could be mutated thereafter its signaling can be assessed following pathogenic insult and/or in the presence of S1P. In addition, it is unclear whether this effect of S1P would be only on CD11b^+^ macrophages, or also in other cell types in the lung. To confirm the action of SPHK2 generated S1P in other cell type it would be required to perform *in vitro* studies using specific cell lines or shorted primary cells and targeted delivery of SPHK2 cDNA in mice following lung injury. How SPHK2-S1P→STING would be regulated in the context of inflammation? The possibility of the regulation of SPHK2 activation and generation of S1P following lung infection is depend upon the generation of NF-κB or generation of cGAMP. Answer to all of these questions need critical experiment design and execution in future.

Taken together, our findings suggest that during acute lung injury, CD11b^+^Mφ in the air space can educate AMφ to resolve ALI through suppressing STING signaling. Our study showed that inhibition of SPHK1 expression in SPHK2 null BMDM had no effect on STING activity [7].

Our finding also showed that SPHK2 regulate STING distribution to Golgi body following its activation, while inhibition of SPHK2 promoted STING interaction with TBK1, leading to its activation. Further studies showed that S1P formed a complex with STING in LPS treated bone-marrow derived macrophages (BMDM). We further showed S1P inhibited cGAMP induced IFN-β production in SPHK2-null BMDM nearly to the levels of WT-BMDM, indicating SPHK2-induced S1P binds STING and inhibits cGAMP activation of STING inflammatory signaling [7] (Figure 1). cGAMP interacts with downstream of SPHK2-mediated S1P generation. These findings could be the basis for future study where SPHK2 generated S1P in CD11b^+^ macrophages may be a targeted delivery option for the therapeutics of lung inflammatory injury.
